# Maximized nanodrug-loaded mesenchymal stem cells by a dual drug-loaded mode for the systemic treatment of metastatic lung cancer

**DOI:** 10.1080/10717544.2017.1375580

**Published:** 2017-09-18

**Authors:** Sen Yao, Xuqian Li, Jingxuan Liu, Yuqing Sun, Zhuanhe Wang, Yanyan Jiang

**Affiliations:** Key Laboratory of Smart Drug Delivery, Ministry of Education and PLA, Department of Pharmaceutics, School of Pharmacy, Fudan University, Shanghai, China

**Keywords:** Tumor-targeted therapy, mesenchymal stem cells, membrane modification, doxorubicin, biotin, avidin

## Abstract

Mesenchymal stem cells (MSCs), exhibiting tumor-tropic and migratory potential, can serve as cellular carriers to improve the effectiveness of anticancer agents. However, several challenges, such as the safety issue, the limited drug loading, the conservation of stemness and migration of MSCs, still remain in the MSC-based delivery system. In the present study, a novel nano-engineered MSC delivery system was established by loading doxorubicin (DOX)–polymer conjugates for the systemic treatment of pulmonary metastasis of breast cancer. For the first time, a dual drug-loaded mode, endocytosis and membrane-bound, was adopted to achieve the maximum amount of DOX conjugates in MSCs. The *in vitro* studies revealed the loaded MSCs possessed multifunctional properties, including preservation of the stemness and migration of MSCs, excellent stability of drug loading, acid sensitive drug release and obvious cytotoxicity against 4T1 cells. The *in vivo* studies confirmed that the loaded MSCs mainly located and long stayed in the lung where the foci of metastatic tumor situated. Importantly, loaded MSCs can significantly inhibit the tumor growth and prolong the life span of tumor-bearing mice in contrast with DOX and DOX-conjugate. The present loaded MSCs system suggested a promising strategy to solve several issues existed in cell-based delivery systems. Especially for the problem of low drug loading, the strategy, simultaneously loading nanodrug in cells’ internal and membrane, might be the most desirable method so far and could be developed as a generalizable manner for cell-mediated tumor-targeted therapy.

## Introduction

Mesenchymal stem cells (MSCs), exhibiting preferable tumor-tropic and migratory properties (Gao et al., 2013; Sohni & Verfaillie, 2013; Sadhukha et al., 2014), over the past few years, have been extensively studied and discussed as cancer therapy vehicles for gene (Amara et al., 2014; Uchibori et al., 2014), small molecule drugs (Augusto et al., 2011) and nanodrugs (Huang et al., 2013; Sadhukha et al., 2014). Basically, two methods, spontaneous endocytosis and cell membrane anchoring, are used to load anticancer agents in MSCs. Cargoes incorporated MSCs by endocytosis can reduce immune clearance and change the toxicity profiles, but also serve as a drug reservoir to sustain release drugs in tumor site (Fligh, 2012; Auffinger, 2013; Hashemi & Kalalinia, 2015). However, sometimes the encapsulation had toxicity to MSCs and affects the intrinsic functions of MSCs (Hashemi & Kalalinia, 2015). On the other hand, a nanodrug (nanoparticles, liposomes or conjugates) can anchor onto the cell membrane of MSCs. The membrane-engineered method can be classified into three categories, insertion or fusion by lipid, biomarker-mediated and chemical modification. For example, a lipid–heparin conjugate was inserted in MSC membrane to change the distribution of MSCs *in vivo* (Kim & Tae, 2015). A CD90 antibody DOX nanorattle was bioconjugated on MSC membrane to treat the glioma (Li et al., 2011). A peptide was covalent bound on cell surface to control the cell–microenvironment interactions (Cheng et al., 2012). Drugs fixed to cell membrane surface have no or ignorable cytotoxicity to MSCs, while the prerequisite is to avoid the subsequent internalization of drug into cells (Wang et al., 2015). However, insertion or fusion by lipid and biomarker-mediated membrane anchoring were instability and easily internalized into cytoplasm (Stephan & Irvine, 2011); thus, it is difficult to obtain a high drug loading due to the limited intracellular space. In contrast, chemical modification exerted stronger binding so that nanosized particles may locate on cell surface long enough to permit MSC migration into target tissues (Stephan et al., 2010).

In our previous study (Zhu et al., 2010; Zhang et al., 2015), the doxorubicin (DOX) conjugate as a nanodrug, PEG-PAMAM-cis-aconityl-DOX (PPCD), obtained by conjugating DOX to PEGylated PAMAM dendrimer via acid-sensitive linkage, was loaded in MSCs by simple endocytosis as a ‘Trojan Horse.’ After intracranial administration of the Trojan Horse, orthotopic glioma-bearing mice acquired a longer survival time. Nevertheless, it still remained a limitation which DOX content in MSCs was insufficient to meet the effective dose of systemic administration in the security cell number.

For the first time, a maximized nanodrug-loaded MSC delivery system, carrying the DOX conjugates by a dual drug-loaded mode, endocytosis accompanied by cell surface anchoring, was constructed in the present study. Considering the high affinity between biotin and avidin (Lesch et al., 2010) which was the strongest non-covalent affinities known, at least 10,000 times stronger than the interaction between antibody and antigen, biotin was chose to modify MSC membrane and DOX conjugates, respectively, and avidin was used as a bridge of DOX conjugates and MSCs. Biotinylated PPCD (BPCD) conjugates were firstly conveyed into MSCs by spontaneous incorporation and subsequently anchored on biotinylated membrane of MSCs via avidin. The nanodrug-loaded MSC system could carry therapeutic dose for systemic administration. Based on the discovery of great tropism of MSCs toward lung tumors (Hong et al., 2009; Yan et al., 2016), the inhibition efficacy of lung metastases of breast cancer was performed in 4T1-bearing mouse models. It is expected that the loaded MSCs can first accumulate in lung after intravenous injection and mediate BPCD to target metastases. The loaded cargo could be liberated from MSCs into tumor niche in the different manners. The membrane-anchored BPCD could either release free DOX in response to the tumor acidic environment or competitive binding with biotin receptor which was higher expressed on 4T1 tumor cells (Shi et al., 2014; Nateghian et al., 2016). On the other hand, the intracellular drugs of MSCs, including DOX conjugates and free DOX gradually degraded from DOX conjugates, were exocytosed by MSCs via the passive diffusion or bumped by P-glycoprotein (Supplementary Scheme S1). To validate the hypotheses, the related studies, including release trend, stability, effect of drug loading on MSC adhesion, proliferation and differentiation, tumor migratory and antitumor efficacy, were conducted *in vitro* and *in vivo*.

## Materials and methods

### Materials

Avidin was obtained from Sigma-Aldrich (Darmstadt, Germany). Sulfo-NHS-LC-biotin (SNLB) and agarose were supplied by Thermo Fisher Scientific (Waltham, MA). CMFDA-SE and DiR was from Beyotime Biotechnology (Lianyun Gang, China). MSCs derived from bone marrow of SD rats were purchased from Cyagen (CA) and propagated between two and eight generations in MSC culture medium to maintain the stem characteristics of MSCs. 4T1 cell line was from ATCC (MD). Female BALB/c mice were from Shanghai SLAC Laboratory Animal Co., Ltd. (Shanghai, China).

### Construction of drug-loaded MSCs

For maximizing the loading amount of drug, four conjugates in two types (iRGD-/BPCD_І_ and iRGD-/BPCD_П_) were synthesized and characterized (Figure S1 and Table S1). The nanodrug-loaded MSCs by a dual drug-loaded manner were designed by cellular internalization of iRGD-/BPCD_І_ followed by cell surface anchorage of iRGD-/BPCD_П_. Briefly, MSCs were firstly incubated with iRGD-/BPCD_І_ (50 μg/mL, DOX-equiv.) for 12 h. Then, 1 million cargo-endocytosed MSCs were suspended in 1 mL 1 mg/mL of SNLB solution for 30 min with occasional mixing. Unreacted SNLB was removed by centrifugation and washing using PBS contained 100 mM glycine. Biotinylated MSCs were further avidinylated by suspending in 10 μg/mL avidin solution. After blending for 10 min, avidinylated MSCs were separated from the avidin solution and resuspended in iRGD-/BPCD_П_ solution (50 μg/mL, DOX-equiv.) for 10 min. Uncombined iRGD-/BPCD_П_ was removed by centrifugation.

### Drug release from loaded MSCs

4 × 10^5^ loaded MSCs were plated in the supernatant chamber of Transwell (pore size =0.4 µm) which was filled in advance with culture medium (pH 7.4) both supernatant (0.4 mL) and subnatant (0.5 mL) chamber. After 10 h of incubation, the conditioned cultured media (CM) of both supernatant and subnatant well were gathered and gently replaced by fresh medium, this operation was repeated from 10 to 168 h. The total DOX concentration of CM was measured by high-performance liquid chromatography (HPLC) and the viability of loaded MSCs was determined by MTT assay at the last time point. In a separate experiment, culture medium at pH 6.0 was adopted to test the acid-sensitive drug release.

### Stability of DOX conjugates on MSC surface

MSCs loading with iRGD-/BPCD_П_ only in membrane-bound manner were seeded on 35-mm glass culture dishes and imaged at 0 h using confocal laser scanning microscope (Zeiss LSM 710). Then, MSCs with fresh culture medium were incubated continuously and visualized at day 1, day 3, day 5 and day 7, respectively.

### Effects of drug loading on the viability of MSCs

The effect of loading process on MSC viability was evaluated in three different loading manners, including group 1 (endocytosis), group 2 (membrane binding) and group 3 (endocytosis and membrane binding). 5000 MSCs in 200 μL culture medium were planted in one well of 96-well plates. After cultured for 24 h, the media of group 1 and group 3 were replaced by 200 μL iRGD-/BPCD_І_ solution (50 μg/mL, DOX-equiv.). Meantime, group 2 was replaced by fresh culture medium. After incubation for another 12 h, group 2 and group 3 were treated to anchor iRGD-/BPCD_П_ on MSC surface as described above. Unloaded MSCs were set as control. MSC vitality in different group was evaluated through MTT method.

### Effects of drug loading on adhesion and proliferation of MSCs

MSCs with or without drugs were seeded in 96-well plates at a density of 5000 cells per well; half of them was treated by MTT assay to calculate adherent cell number after incubation for 6 h, while the remaining half was treated by MTT assay followed by cultured for another 7 days.

### Multidifferentiation capacity

2 × 10^5^ MSCs were seeded in one well of 6-well plates which were coated with 0.1% gelatin in advance. For osteogenic differentiation, the culture medium was replaced by osteogenic differentiation medium until MSCs grown to 60–70% confluence and refreshed every 3 days. For adipogenic differentiation, as MSCs grown to over-confluence, the culture medium was alternately replaced by adipogenic differentiation medium A (cultured for 3 days) and adipogenic differentiation medium B (cultured for 1 day). The induced differentiation process was terminated after the calcium deposits and neutral lipid vacuoles evidently presented. Cells were stained with alizarin red and Oil Red O to visualize osteogenic and adipogenic differentiation, respectively, after treated with 4% paraformaldehyde for 30 min. MSCs grown in normal culture medium were set as negative groups. Images of the cells were captured by an inverted light microscope (Leica DMI4000D), at 100× magnification.

### Migration potential

The effect of drug loading on MSC migration ability was firstly evaluated in scratch assay using a 2-chamber Culture-Insert (Ibidi, USA). 2.5 × 10^4^ MSCs and 2 × 10^4^ 4T1 cells were, respectively, planted into the left and right chambers of a 2-chamber Culture-Insert placed in 6-well plate. After the cells were confluent in their respective chambers, the Culture-Insert was removed, leaving an ∼500 µm cell-free gap between MSCs and 4T1 cell line. Cells were then cultured by serum-free medium (0 h). Images were taken over the course of 20 h using an inverted fluorescence microscope, at 100× magnification. Both left and right chambers were seeded with 4T1 or MSCs as control groups.

In addition, 24-well Transwell chambers (pore size =8 µm) were employed to investigate the effect of drug loading on MSC migratory number. 2 × 10^5^ MSCs in 200 μL serum-free medium were added to the supernatant chamber of the Transwell. The subnatant chamber was abounded with 600 μL conditioned serum-free medium of 4T1 cells (4T1-CM) or fresh serum-free medium. After co-cultured for 12 h, the cells remained on the interior of supernatant well were gently wiped, whereas the cells, adhered at the undersurface of supernatant chamber, were fixed with 4% paraformaldehyde. Next, the adhered cells were stained with DAPI (1 μg/mL) for 10 min for labeling the nucleus, followed by imaging by an inverted fluorescence microscope at 100× magnification. The number of migrating cells was counted and averaged by 10 randomly selected fields.

### Tumor spheroid penetration

A three-dimensional tumor spheroid model of 4T1 cells in 48-well plate was used to evaluate the penetrability of MSCs; 2000 burdened or unburdened MSCs were added along the edge of 48-well plate (20 μL/well). After co-cultured for 4 h, half of them were fixed with 4% paraformaldehyde for 30 min, followed by confocal microscopy z-axis scanning. The remaining half was continued co-culture for another 8 h and then treated with the same process. The penetrative depth of MSCs into the 4T1 tumor spheroids was measured by Zeiss software.

### *In vitro* inhibition effect on tumor growth

The toxicity of loaded MSCs to 4T1 was executed by co-culture experiments in 24-well Transwell chamber (pore size =0.4 µm). 4T1 cells were plated in the subnatant well and cultured for 24 h. Then loaded and unloaded MSCs were seeded in the supernatant well at ratio of 1:1 or 1:10 (MSCs: tumor cells). Four days later, the supernatant chamber was removed and the viability of MSCs and 4T1 was performed by MTT assay.

### *In vivo* studies

The study protocol was performed in compliance with the guidelines of the Ethics Committee of Animal Center of Fudan University. BALB/c mice (female, 6–8 weeks old) were given 5 × 10^5^ 4T1 cells by intravenous injection to establish lung metastasis models of breast cancer.

To ascertain the location of MSCs *in vivo*, 10^6^ DiR-labeled MSCs or BPCD/MSCs were injected via tail vein at day 6 after 4T1 transplantation in mice and imagined by Perkin Elmer IVIS Spectrum live animal imager (IVIS, Waltham, MA), the resulting fluorescence was monitored over the course of 15 days. In the meantime, three mice in each group were sacrificed at 24 h after injection, and the biodistribution of injected MSCs was analyzed by measuring the fluorescence intensity of DiR in brain, heart, kidney, spleen, liver and lung using IVIS. To investigate the location of MSCs in the lung and the tumor tropism of MSCs, MSCs were labeled with CMFDA-SE. After 24 hours of systemic administration, lungs were removed, and histological sections were stained with DAPI and observed by inverted fluorescence microscope.

The tumor-bearing mice were randomly divided into five groups (*n* = 11) and intravenously injected with saline, free DOX, MSCs, BPCD and BPCD/MSCs, respectively, at the dose of 1 mg DOX-equiv./kg at 6, 9, 12, 15 and 18 days after tumor cell transplantation. At day 21 post-transplantation, three mice in each group were sacrificed. The lung was immediately immersed in 4% paraformaldehyde and then treated by H&E and TUNEL.

### Statistical analysis

All the values were presented as mean ± standard deviation (SD) and comparison among the different groups was performed by one-way ANOVA, and *t*-test was used to analyze the data. Kaplan–Meier method was used to compare the life span of individual groups. The level of statistical significance in all statistical analyses was set at a probability of *p < .05.

## Results

### Construction and characterization of loaded MSCs

**Figure 1.**
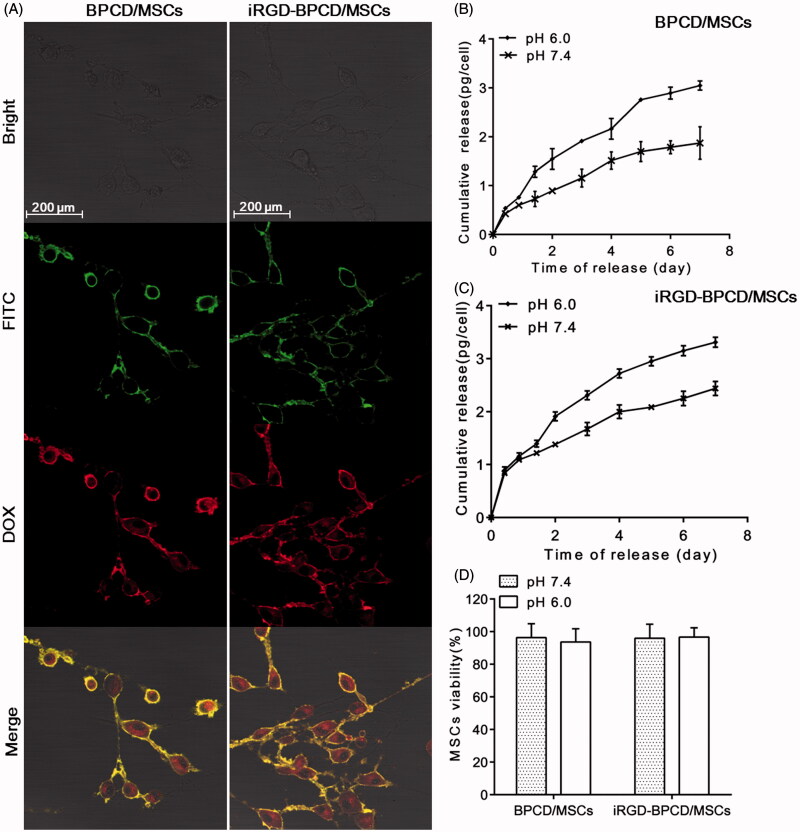
Loading of DOX conjugates into MSCs and drug release. (A) Morphology of BPCD/MSCs and iRGD-BPCD/MSCs under confocal microscope (green fluorescence represented FITC-labeled avidin, red fluorescence represented DOX, and yellow fluorescence indicated the co-localization of FITC and DOX). (B,C) Drug release curves of loaded MSCs under pH 7.4 and 6.0. (D) MSC viability after drug release. The results are presented as the mean ± SD (*n* = 3).

For enhancing the drug loading of MSCs, both types of iRGD-/BPCD with different modification degree were obtained by controlling the reaction molar ratio and reaction time. The positive charge iRGD-/BPCD_І_ conjugates with fewer modified degree were favorable for endocytosis and used to spontaneously incorporate by MSCs, while the negative iRGD-/BPCD_П_ conjugates were bound onto cell membrane through the biotin–avidin linker. The construction of BPCD/MSCs and iRGD-BPCD/MSCs was visualized via confocal microscopy. As shown in Figure 1(A), FITC–avidin with green fluorescence mainly concentrated on MSC membrane surface, while the red fluorescence from DOX conjugates was observed not only on cell surface but also in cytoplasm. After overlapping the imaging pictures, yellow fluorescence appeared on MSC membrane indicated the co-localization of FITC–avidin and DOX conjugates, but cellular interior is still red. The phenomena confirmed that MSCs could load DOX conjugates in dual manner of endocytosis and membrane binding. By determining the DOX amount before and after incubating with MSCs via HPLC, the average endocytosis amounts were 8.4 pg/cell for BPCD_І_ and 7.25 pg/cell for iRGD-BPCD_І_, while each MSC simultaneously anchored 13.03 pg of BPCD_П_ and 11.19 pg of iRGD-BPCD_П_ on cell surface, respectively. These results also coincided with the phenomenon shown in Figure 1(A) in which the DOX fluorescence intensity was stronger on membrane than in cytoplasm. The total amounts of BPCD/MSCs and iRGD-BPCD/MSCs via detecting the DOX concentration in the stem cell lysates were 20.59 pg/cell and 18.24 pg/cell, respectively (Supplementary Table S2).

### Drug release from stem cells and stability of DOX conjugates on MSC surface

In pH 7.4 and pH 6.0 medium, the accumulative amount of DOX liberated from BPCD/MSCs within 7 days was 1.87 pg/cell and 3.05 pg/cell, and that of iRGD-BPCD/MSCs was 2.44 pg/cell and 3.31 pg/cell, respectively (Figure 1(B,C); Supplementary Table S2). The enhancing release amount of DOX in the lower pH medium confirmed the acid-sensitive response of BPCD/MSCs and iRGD-BPCD/MSCs. Combining the total amount of DOX conjugates loaded by MSCs, 62.9% increasing of cumulative release of BPCD/MSCs were obviously higher than 35.7% of iRGD-BPCD/MSCs with decreasing pH, because BPCD/MSCs exhibited less release in pH 7.4 than iRGD-BPCD/MSCs. After undergoing a 7-day release, MSCs still maintain high cell viability in both pH 7.4 and pH 6.0 (Figure 1(D)). The results clearly stated that the increased drug release was not caused by the death of MSCs and also denoted the loading drugs had no toxicity to MSCs during drug release.

**Figure 2.**
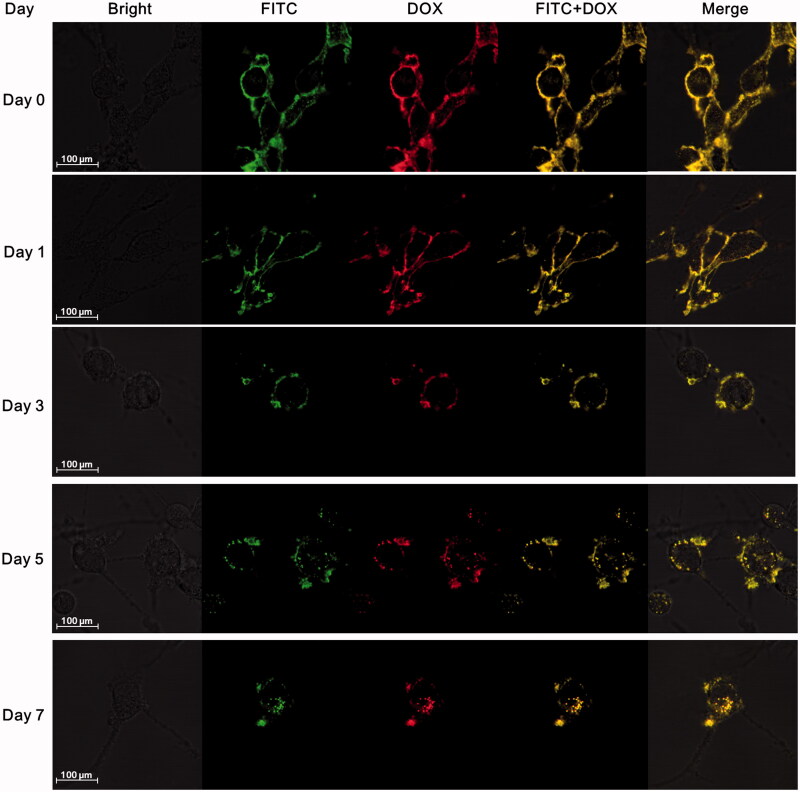
Stability of BPCD_П_ on MSC surface (green fluorescence represented FITC-labeled avidin, red fluorescence represented DOX, and yellow fluorescence indicated the co-localization of FITC and DOX).

The anchoring time of DOX conjugates on the surface of MSCs was also detected by confocal microscopy over time (Figure 2; Supplementary Figure S2). Nearly all DOX conjugates were initially localized on MSC surface, only a tiny bit distributed in cytoplasm. No obvious endocytosis happened and most of DOX conjugates still remained on membrane till day 3, while a weak yellow fluorescence was observed in cytoplasm at day 5 and the intensity gradually increased from day 5 to day 7. Although endocytosis was existed, there is still considerable drug amount to anchor on MSC membrane within 5 days.

### Effects of nanodrugs loading on the viability, adhesion, proliferation and differentiation of MSCs

**Figure 3.**
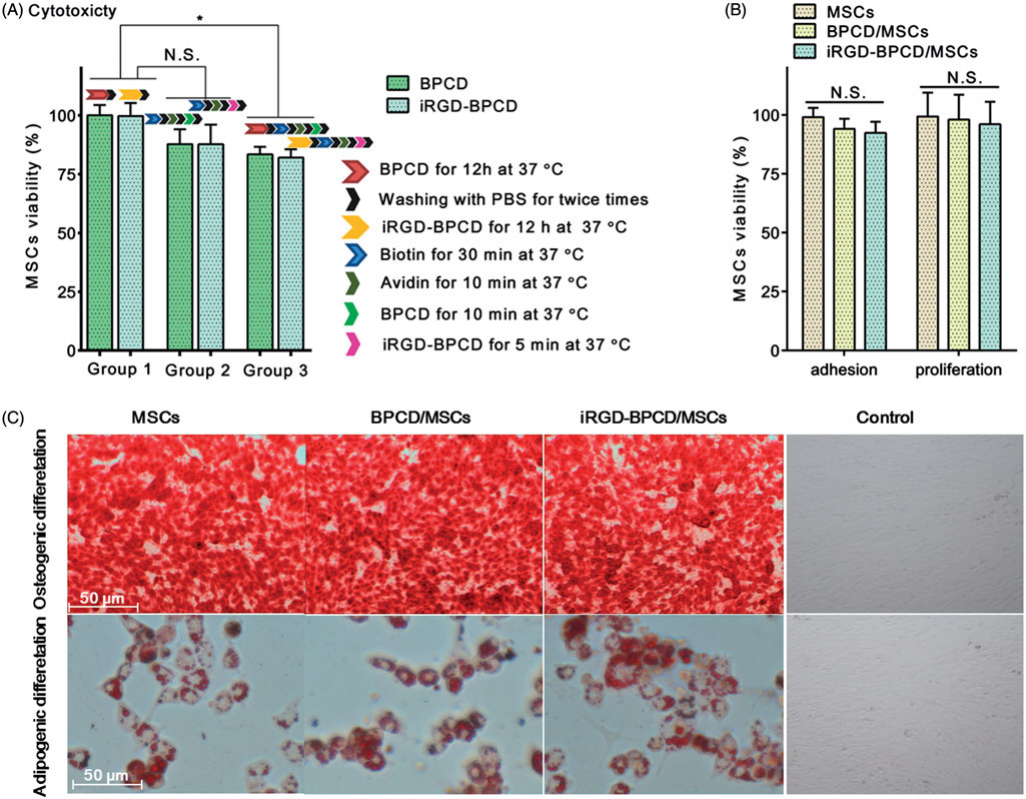
Characterization of drug-loaded MSCs. (A) The effect of drug loading process on the MSC viability, group 1: only endocytosis; group 2: only membrane binding; and group 3: combination of endocytosis and membrane binding. (B) The effect of drug loading on MSC adhesion and proliferation. (C) Osteogenic and adipogenic differentiation of BPCD/MSCs and iRGD-BPCD/MSCs; calcium deposits were stained red by alizarin red in osteogenesis, and neutral lipid vacuoles were stained dark red by Oil Red O in adipogenesis. MSCs cultured in regular medium were set as control. The results are presented as the mean ± SD (*n* = 6). *p < .05; N.S.: not significant.

The influence of different loading manners on MSC viability was evaluated (Figure 3(A)). The internalization procedure of iRGD-/BPCD_І_ had no toxicity to MSCs, and the membrane binding processing of iRGD-/BPCD_П_ had slight effect on MSC viability but no significant difference. However, there was a statistical decrease in viability when MSCs were treated in turn by endocytosis and membrane binding. Even so, the remaining viability was still more than 80% which basically meet the requirement of MSC delivery system. Generally, normal MSCs cultured *in vitro* can adhere to the wall in 6 hours and proliferate after 30 hours. In the present study, the adhesion of drug-loaded MSCs was compared with unloaded MSCs via measuring the viability of adherent MSCs. After culturing for 6 h, no significant decrease in the viability of loaded MSCs indicated the loaded DOX conjugates did not influence the adhesion of MSCs (Figure 3(B)). The long-term effects of DOX conjugates loading on MSCs were investigated via the proliferation assay of loaded MSCs after incubating for 7 days. The results displayed the loaded DOX conjugates did not affect the proliferation of MSCs (Figure 3(B)).

The experiments of osteogenic and adipogenic differentiation were performed to evaluate the differentiation potentiality of MSCs (Figure 3(C)). After culturing in different induced differentiation media, loaded MSCs as well as MSCs could form calcium deposits and neutral lipid vacuoles, respectively, confirmed characteristics of osteogenic and adipogenic differentiation.

### *In vitro* migration and tumor spheroid penetration of the loaded MSCs for 4T1 cells

**Figure 4.**
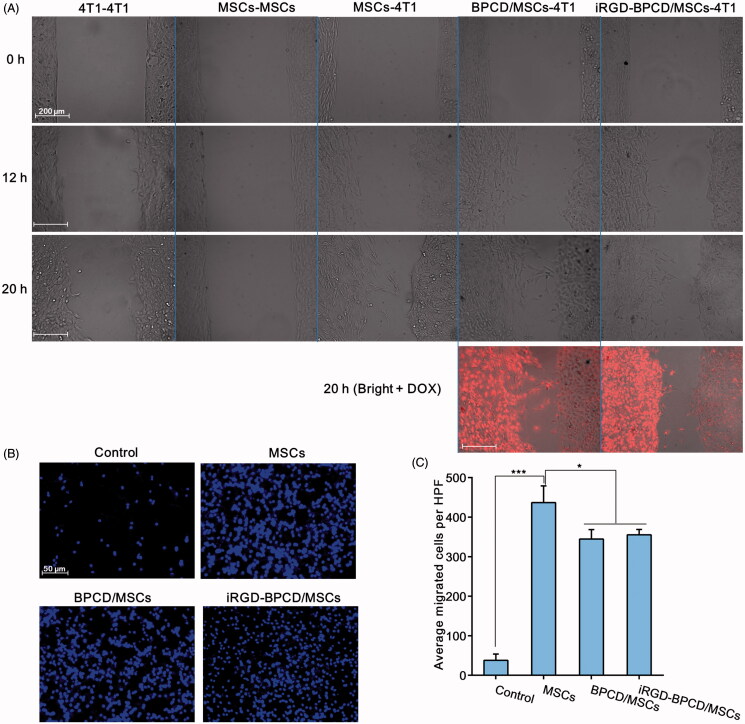
Effect of drug loading on MSC migration. (A) The migration of loaded and unloaded MSCs toward 4T1 clusters, 4T1 toward 4T1 and MSCs toward MSCs was set as control (red fluorescence represented DOX). (B) Microscopic images of loaded and unloaded MSCs migrating through the Transwell membrane toward 4T1-CM, control was MSCs migrated toward fresh serum-free medium, and blue point represented the nucleus of migrated MSCs. (C) The cell amount of the migrated MSCs. HPF was short for high power field. Data were expressed as mean ± standard deviation (*n* = 6). *p < .05, ***p < .001.

The migration ability of MSCs toward 4T1 *in vitro* was first examined by the scratch experiments using 2-chamber Culture-Insert (Figure 4(A)). In order to avoid the interference of MSC proliferation, the experiment time was controlled within 20 hours. The same cells (4T1–4T1 or MSCs–MSCs) were separately seeded in the left and right chambers as control. For the 4T1–4T1 group, both 4T1 cell clusters widened slightly at 12 h and gradually extended toward the middle due to the cell proliferation, but not merged with each other at 20 h. In contrast, there was little change in MSCs–MSCs group in 20-h incubation period. When one side of MSCs was replaced by 4T1 as chemoattractant, the MSC cluster became loose and gradually migrated toward 4T1 clusters until converged with the edge of 4T1 at 20 h. The similar phenomenon was also observed in the loaded MSC group. Obviously, the convergence of MSC clusters and 4T1 clusters mostly attributed to the tropism and migratory nature of MSCs, rather than cell proliferation. Importantly, whether in BPCD/MSCs–4T1 group or iRGD-BPCD/MSCs–4T1 group, red fluorescence from DOX was also detected in the area of 4T1 at 20 h. It was a reasonable speculation that drugs located in 4T1 region could be released from loaded MSCs or still in MSCs.

To further validate the migratory potentiality, Transwell chamber was employed to simulate the chemical gradient of chemokines by adding 4T1-CM in subnatant well. It was found that MSCs in the control group showed few migrating cells toward fresh serum-free medium, whereas MSCs could sense and response to 4T1-CM, moving across the membrane pores to approach the 4T1-CM (Figure 4(B)). The migrating amount of MSCs across the Transwell membrane evidently increased when the fresh serum-free medium was replaced by 4T1-CM, increased by 11 times, 9.1 times and 9.4 times for MSCs, BPCD/MSCs and iRGD-BPCD/MSCs, respectively (Figure 4(C)). As for comparison between loaded and unloaded MSCs, BPCD/MSCs and iRGD-BPCD/MSCs elicited a statistical reduction by 17% in the migrating cells. Even so, there were still considerable advantages over the control group, and the remained migration capacity should be promising and feasible for MSCs mediating DOX conjugates homing to tumor.

Furthermore, the tumor penetration ability of loaded MSCs was examined by 3 D images of 4T1 tumor spheroid (Supplementary Figures S3 and S4). The average penetration depth of MSCs and loaded MSCs were about 59 and 54 μm at 4 h and increased to 90 and 84 μm at 12 h. The results suggested that the penetration was a time-dependent process and the drug loading did not influence the tumor penetrability of stem cells.

### *In vitro* inhibition effect of loaded MSCs on tumor growth

The antitumor effects of BPCD/MSCs and iRGD-BPCD/MSCs against 4T1 cells were verified by using 24-well Transwell chambers (Supplementary Figure S5). After 4 days of incubation, unloaded MSCs did not affect 4T1 cells viability, implied MSCs as cellular carriers were neither to promote tumor growth nor to inhibit tumor growth. By comparison, loaded MSCs can obviously inhibit 4T1 proliferation, and the inhibition was increased with the enhanced MSC number (Supplementary Figure S5A). At the ratio of 1:10 (MSCs:4T1), the survival rates of 4T1 were decreased to 62% (BPCD/MSCs) and 68% (iRGD-BPCD/MSCs), while the ratio increased to 1:1 that of 4T1 reduced to 28% (BPCD/MSCs) and 31% (iRGD-BPCD/MSCs). Besides, in different cell ratios, the remaining viability of loaded MSCs (BPCD/MSCs and iRGD-BPCD/MSCs) after 4 days co-cultured was similar to that of unloaded MSCs (Supplementary Figure S5B), denoted that the activity of loaded MSCs against 4T1 cells was contributed by those released drugs from stem cells rather than the exposed ones resulting from dead MSCs.

### *In vivo* studies

Based on the above results, it was found that iRGD-BPCD/MSCs had no advantages over BPCD/MSCs in loading amount, drug release, tropism, tumor penetration and antitumor activity *in vitro*. Especially, iRGD-BPCD/MSCs exhibited lower drug content and faster release than BPCD/MSCs, and it would bring an issue that the iRGD-BPCD/MSC delivery system loading less drug could be liable to leak more drugs in circulatory system and in turn cause fewer drug into tumor tissue. Therefore, BPCD/MSCs were chosen as the optimized MSC-mediated drug delivery system for further *in vivo* studies.

**Figure 5.**
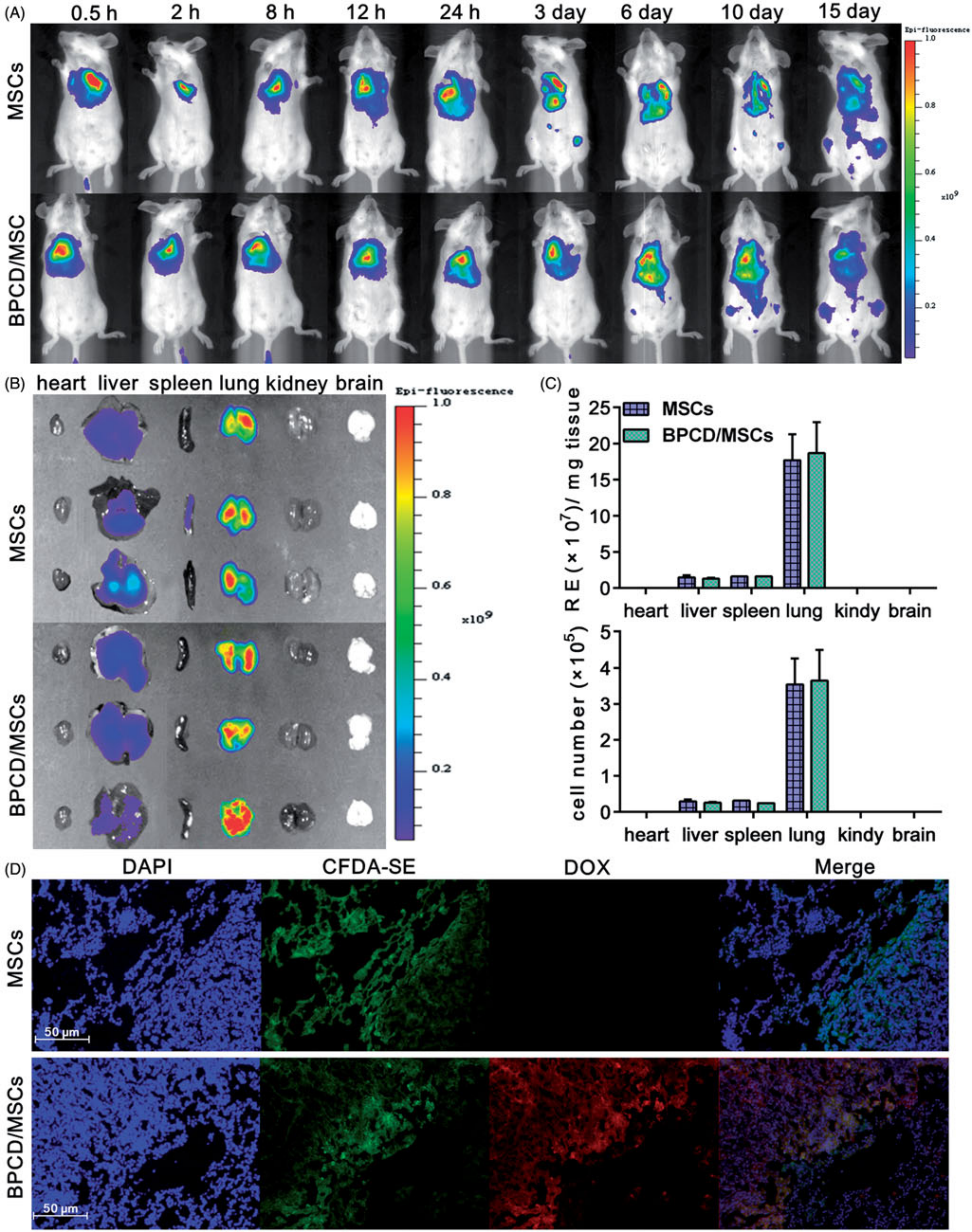
The effect of drug loaded on MSC distribution and retention. (A) The distribution and retention of loaded and unloaded MSCs *in vivo* and MSCs labeled by DIR. (B) *Ex vivo* imaging of major organs harvested from lung tumor-bearing mice 24 h after the intravenous injection of MSCs and BPCD/MSCs. (C) The accumulation of MSCs and BPCD/MSCs in major organs was quantified by the radiant efficiency and cell number (*n* = 6). (D) Microscopic images of histological sections from lung metastasis of breast cancer-bearing mice, blue fluorescence indicated the nuclei of tumor cells, green represented MSCs, red represented DOX, and purple indicated the co-localization of nuclei, MSCs and DOX.

After MSCs labeled with DiR (MSCs-DiR) were intravenously injected into 4T1-bearing mice, its biodistribution behavior was monitored by IVIS. As observed in Figure 5(A), two groups of animals treated by loaded or unloaded MSCs displayed the same distribution behavior. At 0.5 h post-injection, a strong fluorescence was detected in the lungs. Though the fluorescence signal was declined over time and with a slight tendency to migrate to the liver and other organs, most of them were still accumulated in lung and can be detected for at least two weeks. After 24 h post-injection, the fluorescence intensities of major organs were imaged and quantified by the radiant efficiency (Figure 5(B,C)). Almost all detectable fluorescence was concentrated in lung (93%) besides a tiny part accumulated in liver (6.3%) and spleen (0.7%), similar results were also obtained in unloaded MSC group, and there were 85.5, 7 and 7.5% for lung, liver and spleen, respectively. In addition, histological sections of lung metastases were imaged in Figure 5(D), and it was found that loaded and unloaded MSCs mainly distributed in the tumor area. Therefore, the results confirmed that loaded MSCs via systemic administration could rapidly mediate BPCD to accumulate in lung where the tumor located and detained there for a long time.

**Figure 6.**
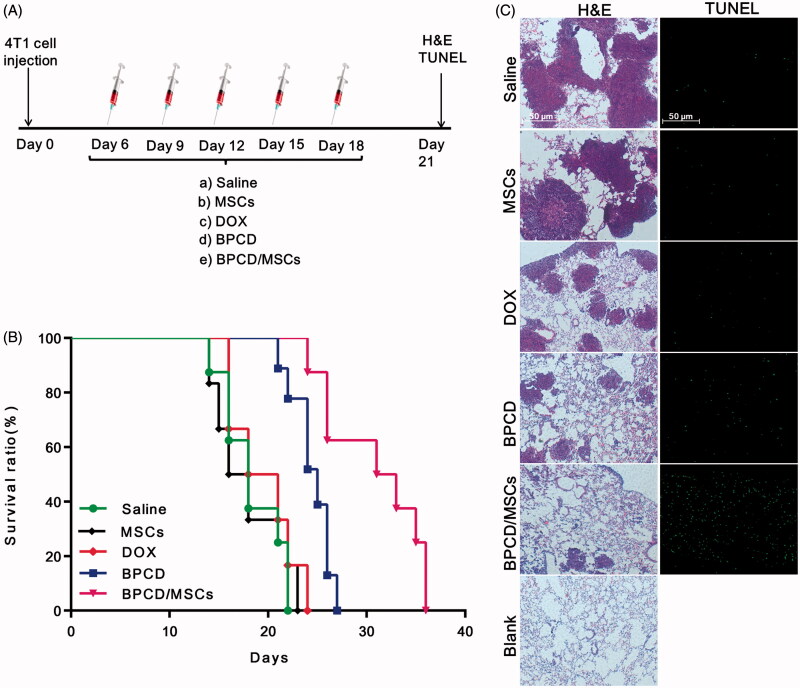
The antitumor effect of BPCD/MSCs *in vivo*. Saline, MSCs, DOX, BPCD and BPCD/MSCs were systemically administrated after tumor implantation. (A) Illustration of the dosage regime. (B) Kaplan–Meier survival curve of lung metastasis of breast cancer-bearing mouse. (C) H&E sections and TUNEL staining of lung tissues from mice for breast cancer lung metastasis (normal mouse lung as blank), the network structures were alveoli, the deeper color region with a great number of cells tightly clustered together was tumor cells, and green represents the apoptotic cells.

*In vivo* antitumor efficacy of BPCD/MSCs on metastatic lung cancer was studied in 4T1-bearing mice. The treatment groups (DOX, BPCD and 10^6^ BPCD/MSCs) and control groups (saline and 10^6^ MSCs) were intravenously administrated once every three days (Figure 6(A)). Apparently, the survival curve of MSCs was almost identical with that of saline (Figure 6(B)), verifying MSCs itself does not affect tumor growth. In contrast, the mice from both BPCD and BPCD/MSC groups revealed significantly longer life span over other three groups. As shown by the log-rank test in Table S3, both BPCD and BPCD/MSCs significantly prolonged the median survival of mice as compared with the control groups, while there was no statistical difference between the DOX group and the control groups. It was noteworthy that the BPCD/MSC treatment group exhibited the best therapeutic effect among all test groups and the increase in life span (ILS) was 26.6% and 58.5% in contrast to BPCD and DOX groups, respectively.

The antitumor activity of BPCD/MSCs was further evaluated at the level of histopathology by H&E and TUNEL staining (Figure 6(C)). For saline group and MSC group, the whole section was nearly occupied by tumor cells and alveoli were less than the normal lung tissue. For treatment groups, the area and number of tumor dramatically decreased, especially in BPCD/MSC group. Moreover, the number of apoptotic cells in treatment groups can be ranked as BPCD/MSCs > BPCD > DOX, and the DOX group resulted in the fewest apoptotic cells as well as the two control groups. Since the unloaded MSC group showed a negligible apoptosis effect, it can be confirmed that the apoptotic cells in the BPCD/MSC group were mainly from tumor cells.

## Discussion

MSC-based delivery systems with potent tumor-homing potential have made them attractive candidates for the targeted delivery of anticancer agents. Nevertheless, several challenges still remain in using MSCs as cell vehicles. First of all, the safe issues of transduction with viral vectors and efficiency problems in non-viral vectors have hindered FDA endorsement (Stuckey & Shah, 2014). Second, the interaction between tumors and MSCs is a controversial issue in whether the effect of MSCs on tumor growth is positive, negative or ignorable (Li et al., 2015; Meleshina et al., 2015). Third, the limited drug loading hampers the treatment of MSCs with therapeutic dose by intravenous injection (Augusto et al., 2011; Gao et al., 2013; Zhang et al., 2015). Finally, the conservation of stemness and migration for engineered MSCs must be taken into consideration. To resolve the aforementioned issues, in the present study, a novel nanodrug-loaded MSC delivery system, bypassing the safety challenge of genetically engineered MSCs, was established by a dual-loaded mode for the systemic treatment of pulmonary metastasis from breast cancer.

At present, no consensus exists on the effects of MSCs may have on the host. Some studies proved MSCs’ inhibition effect on cancer cells, others suggested the promoting tumor development, or even there was no influence on the tumor growth. The various effects of MSCs on tumor in these studies might be a result of the different source of stem cells, the different animal models or the different route of MSC administration (Li et al., 2015). To ensure the interaction of MSCs and 4T1 cells, a pre-experiment *in vivo* was performed by subcutaneously transplanted MSCs and 4T1 on mice (Supplementary Figure S6). There were no obvious differences in the tumor growth curves for MSCs/4T1-bearing mice between the 4T1 group, the 4T1 + MSC group and the 4T1→MSC group during 30 days post-transplant; in the meantime, no tumor growth had been found on the transplant site after injecting 10^6^ MSCs. Together with other results from *in vitro* and *in vivo* antitumor effect of MSCs on 4T1, it was certain that MSCs have neither tumorigenicity nor inhibition tumor growth, indicating that MSCs as a cell carrier are safety in the present study.

A common problem of MSCs as cell carrier is the limited drug loading which result in injecting more of MSCs to ensure that an effective drug dose reaches the target site. However, direct injection (intravascular or intra-arterial injection) of a large number of cells causes complications attributed to microembolism, which may lead to vascular obstruction, stroke and potentially death (Park et al., 2015). Thus, the administration route in our previous work and many other drug-loaded MSC studies had to choose the local injection (Pacioni et al., 2015; Park et al., 2015; Zhang et al., 2015). Unfortunately, the local administration is not suitable for deep-seated tumor or metastasis models, while MSCs administered by intravenous injection, as recently reported, are able to migrate specifically to and integrate into tumor stroma and track microscopic metastasis (Li et al., 2015). To actualize the systemic administration, in the present study, a novel loading strategy was explored in order to acquire a high drug content of loaded MSCs. By optimizing the conditions of loading, the average endocytosis amount of MSCs for BPCD_І_ or iRGD-BPCD_І_ was consistent with our previous results (Zhang et al., 2015), while the content of BPCD_П_ or iRGD-BPCD_П_ anchored on the surface of MSCs was markedly more than that of endocytosis. It was suggested that membrane-binding loading of MSCs via the interaction of biotin and avidin is a more effective method to increase drug content without affecting the endocytosis. Moreover, the present nanodrug-loaded MSCs are considered to be a full-load MSC delivery system because of the maximum amount of endocytosis as much as MSCs can and nearly full coverage of MSC membrane surface by biotin.

After several treatment processes, more than 80% of loaded MSCs were still alive. The final living BPCD/MSCs and iRGD-BPCD/MSCs displayed acid-sensitive drug release to ensure the stability of loaded MSCs in circulatory system, with the addition of the anchoring stability of BPCD and iRGD-BPCD on the MSC surface within 5 days, and it would provide the opportunity for DOX conjugates to response the tumor acidic environment to exert the pH-sensitive release. In addition, the results of the adhesion and proliferation suggested the loaded MSCs with the maximum content of DOX conjugates were still able to maintain the normal growing state of MSCs.

Another concern for the present loaded MSCs was focused on the conservation of MSCs’ stemness and tumor tropism. According to the results of the experiments of differentiation, migration and tumor spheroid penetration, it was identically validated the loaded MSCs reserved MSCs’ stemness and tumor-tropic potential.

Attention should be paid so that the iRGD-BPCD/MSCs did not reveal the anticipative advantages over BPCD/MSCs. iRGD as a tumor-targeting and tumor-penetrating cyclic peptide can enhance the permeability of drug into tumor parenchymal via a tumor-specific and neuropilin-1-dependent manner (Lorena et al., 2015). Based on our previous study (Wang et al., 2014), iRGD-modified PPCD could significantly enhance the antitumor efficacy of the conventional RGD-modified PPCD and unmodified PPCD. Thus, in the present study, iRGD-modified BPCD was loaded in MSCs, and it was expected that iRGD could further increase the targeting efficiency of loaded MSCs. However, the desired results did not achieve through a comprehensive *in vitro* investigation. Despite the cause is unknown, together with the results of our previous another work in which the RGD-PPCD/MSCs obtained by endocytosis manner did not show the evident advantages *in vitro* and *in vivo* over PPCD/MSCs (Zhang et al., 2015), it could be suggested that the active targeting nanomedicines loaded in cell carriers might be an inadvisable strategy.

Since obvious antitumor activity against 4T1 tumor cells, the *in vivo* fate of BPCD/MSCs as the final optimized system was investigated in pulmonic metastasis model of breast cancer. The results of biodistribution, that substantial BPCD/MSCs were concentrated in lungs and most of them located at the tumor area, confirmed the tumor tropism of BPCD/MSCs. Furthermore, according to our previous findings that most of DOX conjugates were accumulated in the reticuloendothelial system (Wang et al., 2014), the outcome implied that the MSC-mediated delivery system can improve the biodistribution of nanomedicines. Besides the massive amount of BPCD/MSCs accumulating in pulmonic metastasis, long-term lung retention of BPCD/MSCs for at least two weeks could serve as a drug reservoir benefiting sustained release of drugs. Consequently, the antitumor efficacy of BPCD/MSCs was remarkably higher than that of BPCD.

## Conclusions

In summary, using a dual drug-loaded mode, we successfully established a MSC-based multifunctional delivery system by integrating the hypoimmunogenic and tumor-tropic feature of MSCs and the control acid-sensitive release advantage of DOX conjugates for the systemic treatment of lung metastasis of breast cancer. The optimized BPCD/MSCs with the maximum content of DOX conjugates reserved MSCs’ stemness and tumor-tropic potential. DOX conjugates can stably anchor on the cell membrane for at least 5 days and still maintain the pH-sensitive release properties. BPCD/MSCs as well as MSCs mainly located in the lung where the foci of metastatic tumor situated, and remained at there for at least two weeks. Moreover, BPCD/MSCs exhibited encouraging antitumor activity both *in vitro* and *in vivo*. These findings suggested that MSCs loaded with nanodrugs by a dual drug-loaded mode could achieve the maximized drug loading and be utilized as a potential therapeutic system to improve the therapeutic effect of lung metastasis.

## Supplementary Material

IDRD_Jiang_et_al_Supplemental_Content.zip
