# Supplementary material for: Maximized nanodrug-loaded mesenchymal stem cells by a dual drug-loaded mode for the systemic treatment of metastatic lung cancer
Source: Drug Deliv. 2017 Sep 18;24(1):1372–83. doi: 10.1080/10717544.2017.1375580 (PMC8241180; doi:10.1080/10717544.2017.1375580)
Supplement: IDRD_Jiang_et_al_Supplemental_Content.zip [file IDRD_A_1375580_SM4571.zip › support information.docx]

**Supplementary figures and legends**

**The description of supplementary figures and legends**

**Suppl. fig. 1: Synthesis and characterization of BPCD and iRGD-modified BPCD**

A, ^1^H-NMR spectrum of Biotin-PEG–PAMAM-PEG-Meo. B, The structure of iRGD-BPCD. C, ^1^H-NMR spectrum of Biotin-PEG–PAMAM-PEG-iRGD. Biotinylated PPCD (BPCD) was synthesized according to the previous synthetic route of PPCD with slight modification (17, 30). Briefly, Biotin-PEG-PAMAM was firstly synthesized by mixed PAMAM （Generation 4 PAMAM dendrimer）and Biotin-PEG-NHS Biotin-polyoxyethylene-succinimidyl carboxymethyl ester (Biotin-PEG-NHS, MW 5069) in borate buffer (pH 9.0) for 3 h with the molar ratio of 1:16. After purified by ultrafiltration, the Biotin-PEG-PAMAM-PEG-Meo conjugate (BPP) was synthesized by the reaction of Biotin-PEG-PAMAM and Methoxy-polyoxyethylene-succinimidyl carboxymethyl ester (Meo-PEG-NHS, MW 5087) with the molar ratio of 1:16 and were characterized by ^1^H NMR spectra (Fig S1A). In order to obtain BPCD, the synthesized cis-aconityl-doxorubicin (CAD) according to previous method was mixed with 1-ethyl-3-(3-dimethylaminopropyl) carbodiimide hydrochloride (1:10, mole ratio) in 0.2 M phosphate buffer (pH 6.0) for 30 min in dark, BPP was added and adjusted the pH to 8.0. The unreacted reactants were separated using a Sephadex G-25 fine column. Two types of BPCD (BPCD_І_ and BPCD_П_) with different drug content was obtained via varied feed ratio (BPP: CAD) and reaction time by which the mole ratio of 1:200 for 36 h achieved BPCD_І_ and that of 1:50 for 12 h was BPCD_П,_ respectively. Furthermore, BPCD_І_ and BPCD_П_ were modified with iRGD to obtain iRGD-BPCD_І_ and iRGD-BPCD_П_ (Fig S1B) according to the following steps. After iRGD (internalizing RGD, CRGDKGPDC) (25 μmol) and N-hydroxysulfosuccinimide-polyoxyethylene-maleimide (NHS-PEG-MAL, MW 5069) (25 μmol) stirring in NaAc-HAc buffer (pH 6.0) for 30s, 1 μmol of Biotin-PEG-PAMAM in borate buffer (pH 9.0) was added and mixed in dark for 12 h. β-mercaptoethanol (250 μmol) was added to quench unreacted maleimide group by continuing the reaction under pH 7.0 for another one hour. iRGD-BPP was obtained by ultrafiltration and were characterized by ^1^H NMR spectra (Fig S1C), then CAD was conjugated to iRGD-BPP in the same method as BPCD. The conjugates were characterized by size, zeta potential and DOX content, DOX content (wt. %) and probable number in each conjugate was determined using HPLC.

**Suppl. fig. 2: Stability of iRGD-BPCD_П_ on MSCs surface**

Green fluorescence represented FITC labeled avidin, red fluorescence represented DOX, yellow fluorescence stood the co-localization of FITC and DOX.

**Suppl. fig. 3: MSCs penetration for 4T1 tumor spheroids.**

4T1 cells were seeded at a destiny of 2000 cells/well in agarose coated 48-well plate and gradually gathered into a spheroid. When the diameter of the tumor sphere reached 400-500 μm, MSCs were added. A, Three-dimensional reconstruction images of 4T1 tumor spheroids. Red and green fluorescence represented DOX and MSCs, co-localization, presented yellow, indicated the penetration of loaded MSCs. B, Depth of penetration into 4T1 tumor spheroids at 4h and 12h measured by Zeiss software. Data were expressed as mean ± standard deviation (n=6). N.S. not significant

**Suppl. fig. 4: The scanned images of loaded and unloaded MSCs penetrated into 4T1 tumor spheroids.**

The pictures were obtained from top to middle of the spheroids per 10 μm (Red fluorescence represented DOX and green fluorescence represented MSCs).

**Suppl. fig. 5: *In vitro* anti-tumor effect of loaded MSCs.**

A, Viability of 4T1 after treated by loaded and unloaded MSCs for 4 days. B, Viability of MSCs after 4 days. C, Schematic diagram of Transwell assay (0.4 μm of pore size). Data were expressed as mean ± standard deviation (n=6).

**Suppl. fig. 6: The effect of MSCs on the tumor volume of 4T1.**

A, Tumor volume of different group on day 30. B, 4T1 tumor growth curve. 4T1 group: 10^6^ 4T1 tumor cells were implanted on the right side breast fat pad of fifth pairs in Babl/c mice; MSCs group: 10^6^ MSCs were implanted on the same site of 4T1 implantation; 4T1+MSCs group: 10^6^ 4T1 tumor cells and 10^6^ MSCs were implanted on the same site of 4T1 implantation; 4T1→MSCs group: 10^6^ 4T1 tumor cells were implanted on the right side breast fat pad of fifth pairs, 10^6^ MSCs were implanted when the 4T1 tumor volume is 100 mm^3^.

**Table S1: Characteristics of DOX-conjugates**

*Conjugated number per PMAMA: The relative amount of Biotin, iRGD and PEG was calculated by 1H-NMR spectra using the proton integration method. The DOX numbers per PMAMA were converted from DOX content (wt.%) in DOX-polymer conjugates.

**Table S2: Drug loading content in MSCs and cumulative drug release within 7 days**

*P＜0.05 (The amount of cumulative release of iRGD-BPCD/MSCs vs BPCD/MSCs)

**Table S3: Median survival of mice of lung metastasis of breast cancer**

Median: the median survival. ILS: increase in life span ((*T/C* - 1) × 100%), where *T* and *C* represent the mean survival time of the treated and control animals, respectively. *P* values: were calculated by using the long-rank (Mantel-Cox) test, **p* < 0.05, ****p* < 0.001.

Supplementary figure 1


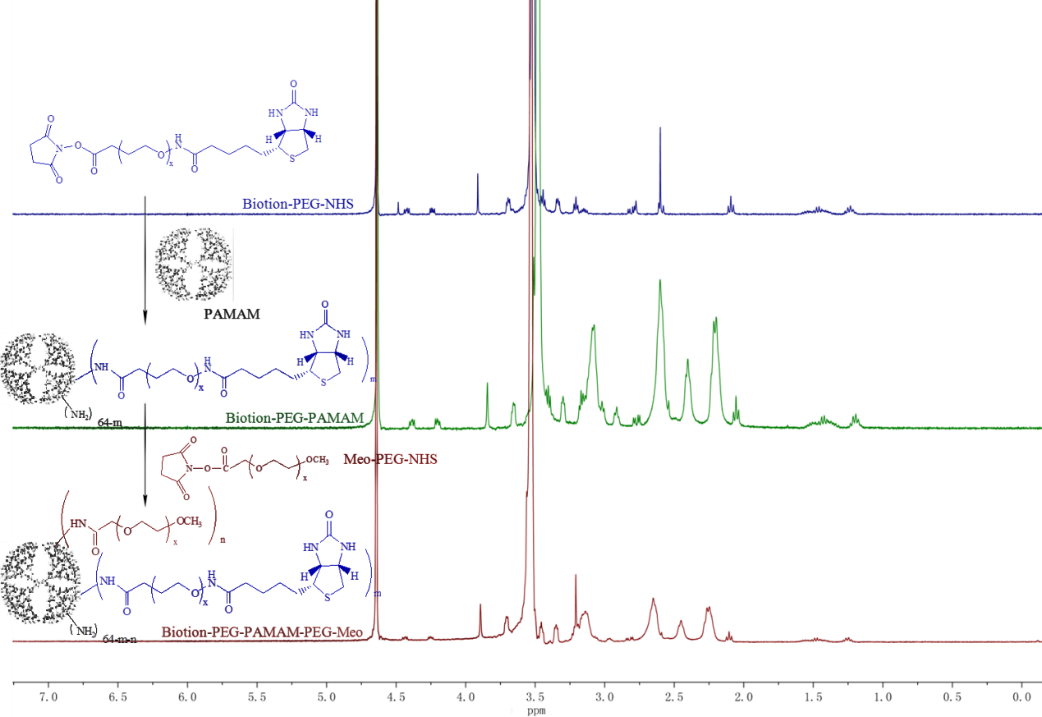


A

B


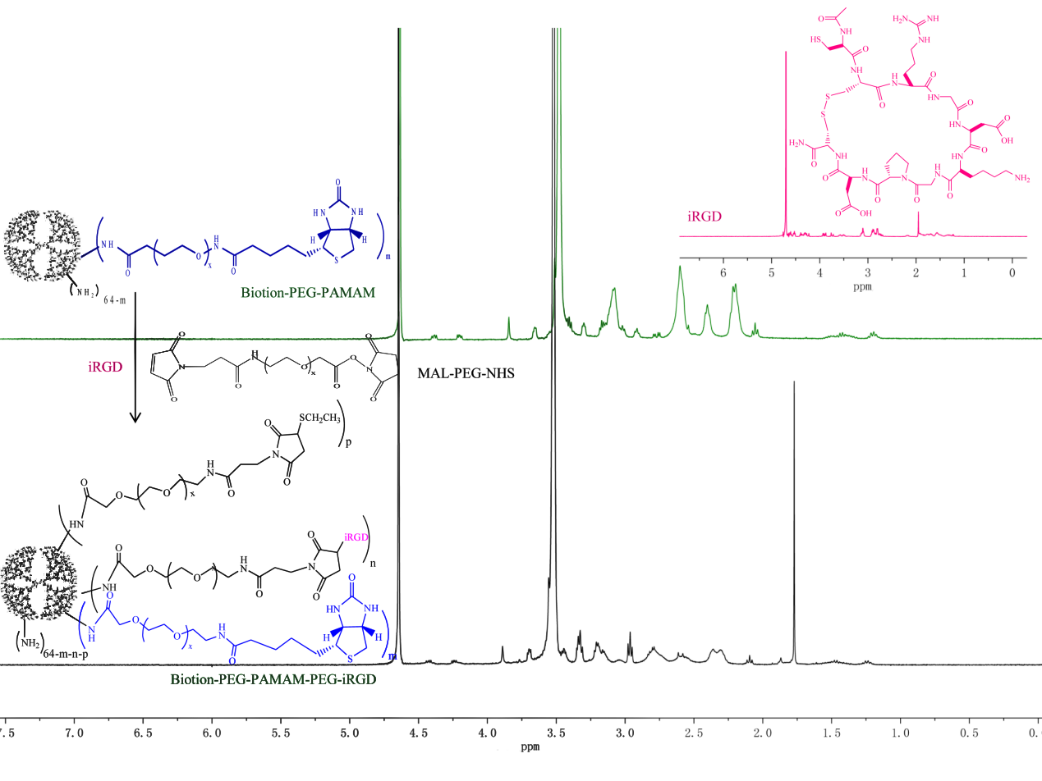


C

Supplementary figure 2


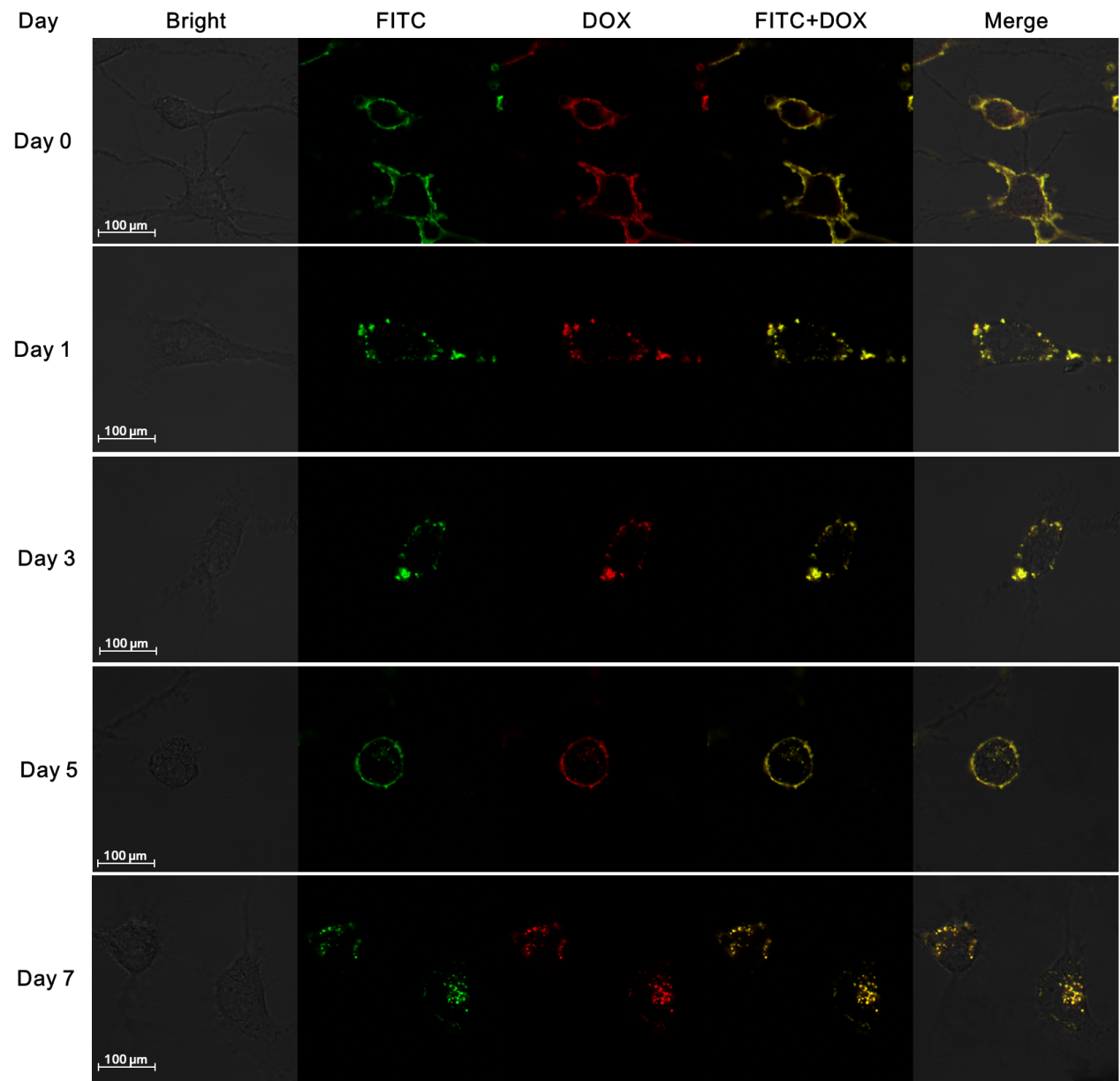


Supplementary figure 3


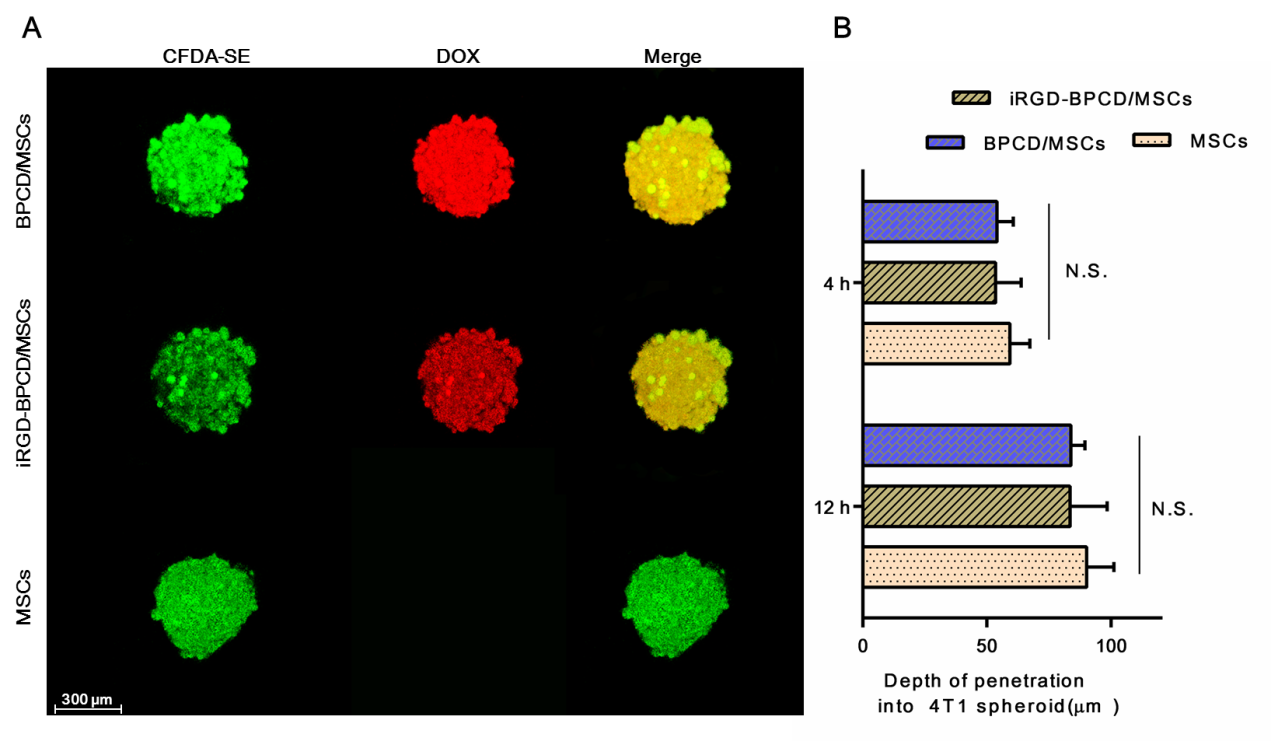


Supplementary figure 4

MSCs


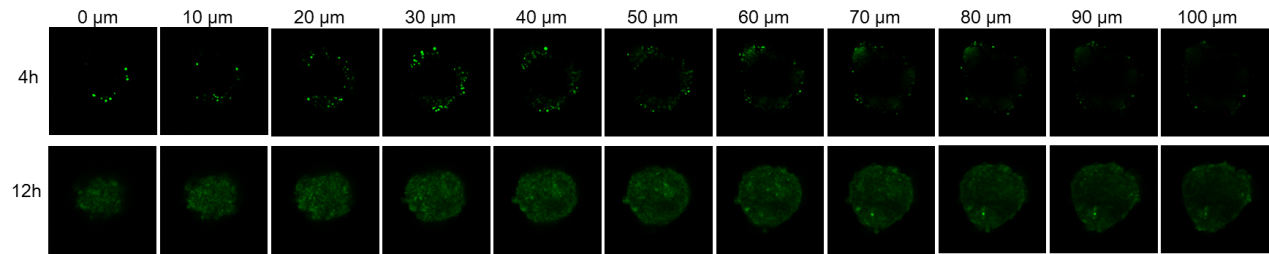


BPCD/MSCs


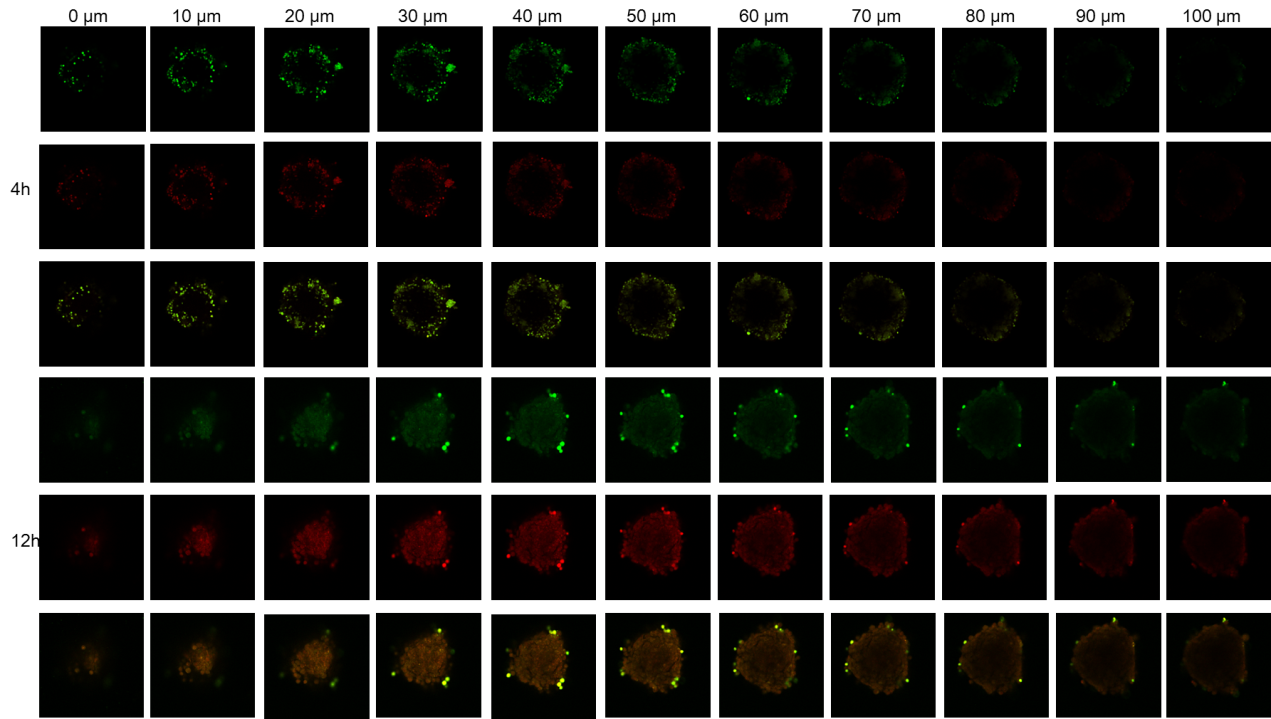


iRGD-BPCD/MSCs


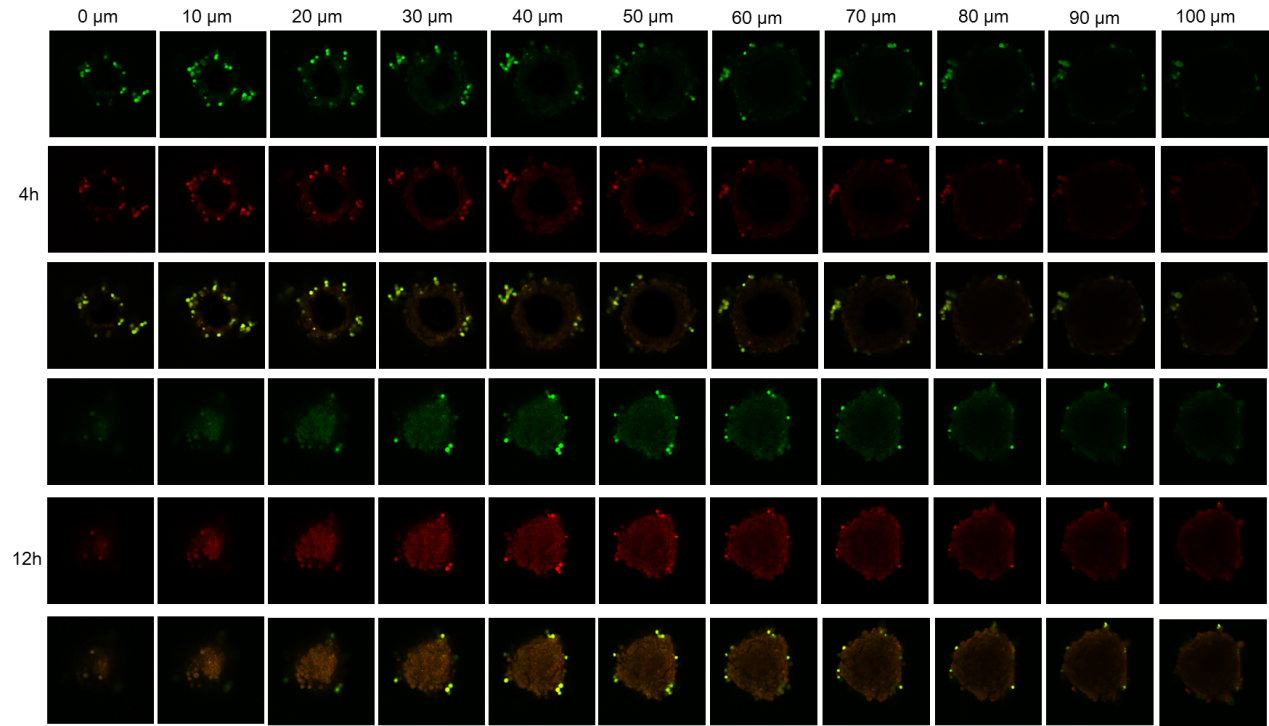


Supplementary figure 5


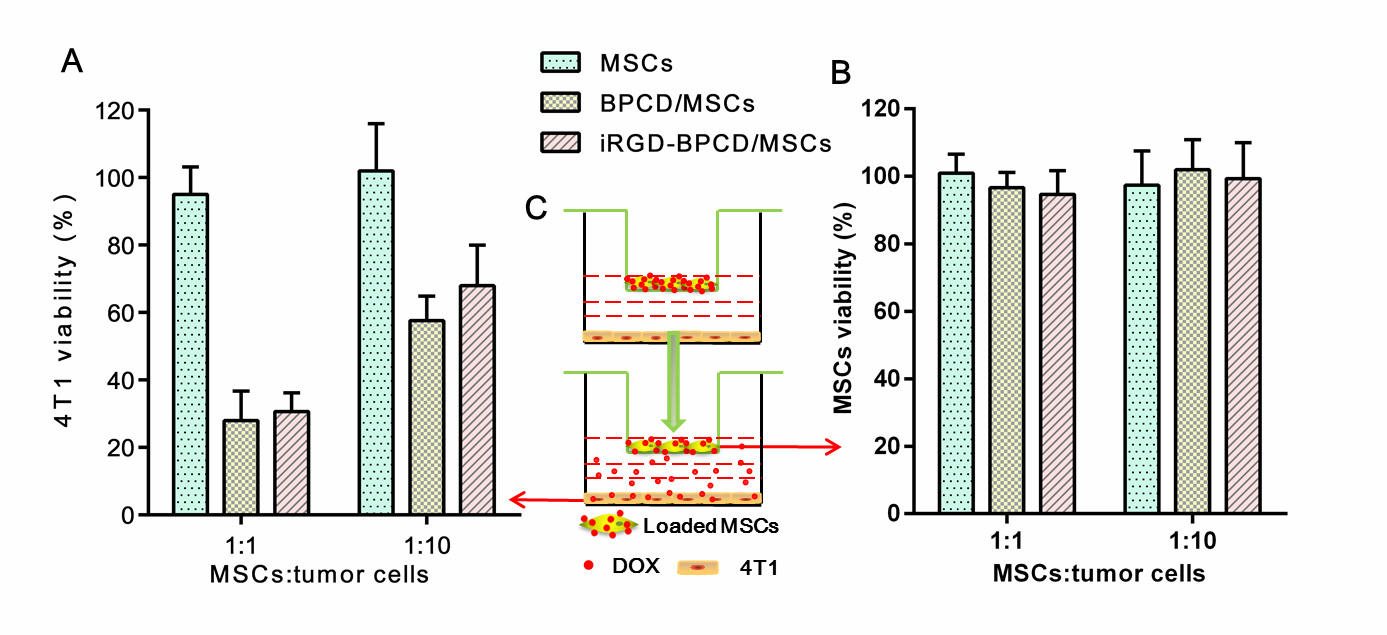


Supplementary figure 6


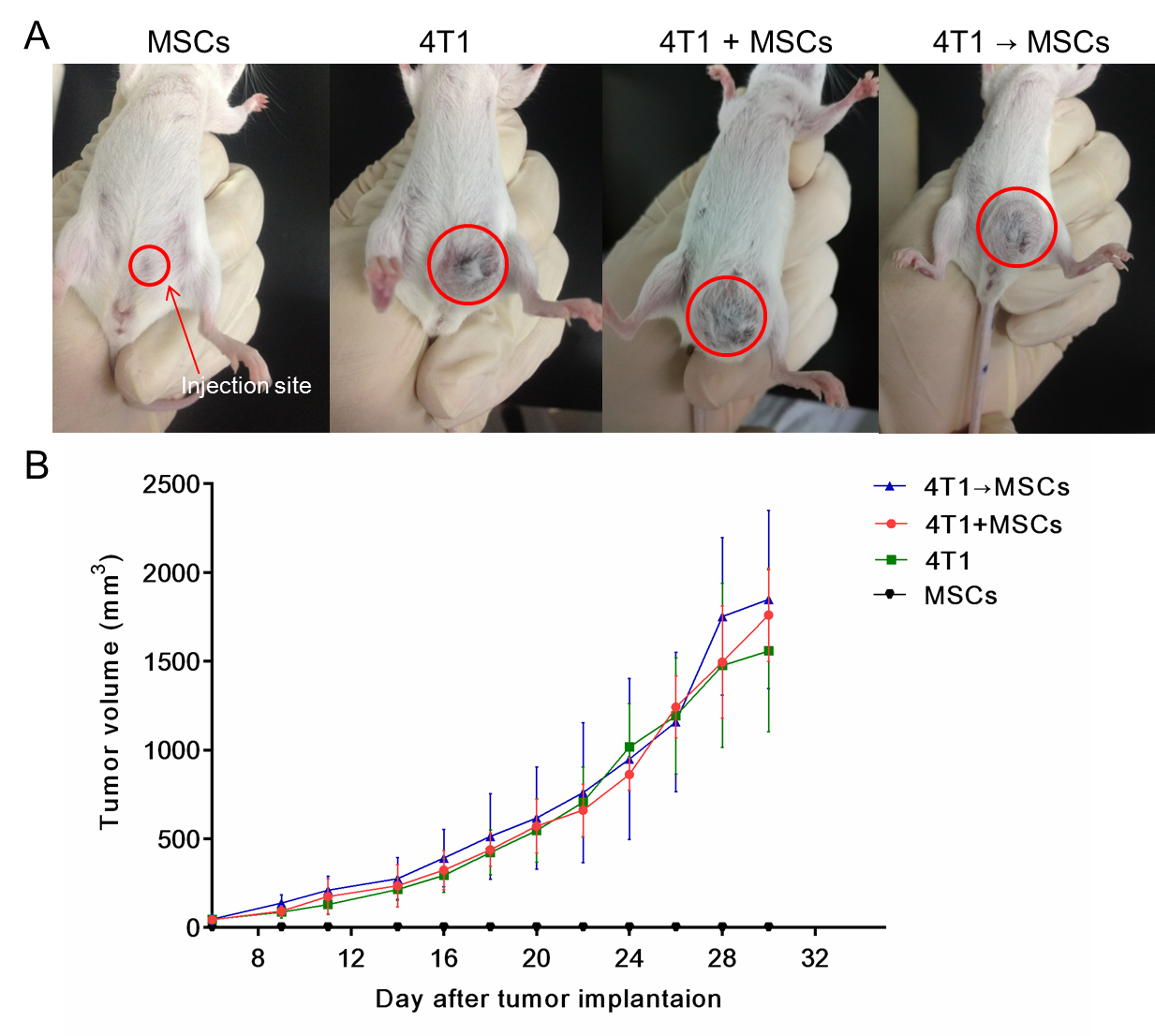


Supplementary table 1

| Conjugates | Conjugated number per PAMAM* | | | | Particle size（nm） | Zeta  Potential  (mV) |
| --- | --- | --- | --- | --- | --- | --- |
|  | DOX | Biotin | Total PEG | iRGD |  |  |
| BPCD_І_  (1/50) | 15.4 ± 1.0 | 8.7 ± 1.7 | 18.9 ±1.8 | / | 48.06 ± 1.37 | 2.69 ± 0.47 |
| BPCD_П_  (1/200) | 35.2 ± 1.3 | 8.7 ± 1.7 | 18.9 ±1.8 | / | 42.68 ± 0.57 | -6.53 ± 0.35 |
| iRGD-BPCD_І_  (1/50) | 13.5 ± 1.2 | 8.7 ± 1.7 | 21.2 ± 1.2 | 7.0 ± 1.0 | 50.09 ± 0.37 | 2.25 ± 0.39 |
| iRGD-BPCD_П_  (1/200) | 37.0 ± 1.1 | 8.7 ± 1.7 | 21.2 ± 1.2 | 7.0 ± 1.0 | 45.71 ± 0.94 | -7.20 ± 1.90 |

Supplementary table 2

|  | BPCD/MSCs | | iRGD-BPCD/MSCs | |
| --- | --- | --- | --- | --- |
| Total C( pg/cell) | 20.59 ± 0.98 | | 18.24 ± 1.26 | |
| Cumulative release( pg/cell) | pH 7.4 | pH 6.0 | pH 7.4 | pH 6.0 |
|  | 1.87 ± 0.18 | 3.05 ± 0.54 | 2.44 ± 0.37^*^ | 3.31 ± 0.48 |
| Release percentage | 9.1% | 14.8% | 13.4% | 18.1% |
| Increase percentage from pH 7.4 to pH 6.0 | 62.9% | | 35.7% | |

Supplementary table 3

| Groups | Median (days) | Mean survival time (days) | ILS (%) | | | |
| --- | --- | --- | --- | --- | --- | --- |
|  |  |  | MSCs | DOX | BPCD | BPCD/MSCs |
| Saline | 18 | 18.4 ± 3.0 | 1.6 | 6.0 | 32.6** | 67.9*** |
| MSCs | 17 | 18.7 ± 3.5 | / | 4.3 | 30.5** | 65.2** |
| DOX | 19.5 | 19.5 ± 3.0 | / | / | 25.1** | 58.5*** |
| BPCD | 25 | 24.4 ± 2.1 | / | / | / | 26.6* |
| BPCD/MSCs | 32 | 30.9 ± 5.0 | / | / | / | / |
