# Supplementary material for: Maximized nanodrug-loaded mesenchymal stem cells by a dual drug-loaded mode for the systemic treatment of metastatic lung cancer
Source: Drug Deliv. 2017 Sep 18;24(1):1372–83. doi: 10.1080/10717544.2017.1375580 (PMC8241180; doi:10.1080/10717544.2017.1375580)
Supplement: IDRD_Jiang_et_al_Supplemental_Content.zip [file IDRD_A_1375580_SM4571.zip › The description of Scheme 1.docx]

**The description of supplementary figures and legends**

**Suppl. scheme 1: Illustration of the construction and tumor targeted delivery of MSCs mediated drug delivery system.**

After two types of DOX-conjugates were synthetized, positive BPCD_І_ conjugates were firstly internalized in MSCs followed by anchoring of negative BPCD_П_ on MSCs surface by the interaction between biotin and avidin. After i.v injection, loaded MSCs can accumulate in tumor, and the loaded cargo could be released from MSCs into tumor niche in the following manners. The membrane-anchored BPCD_П_ were acid-sensitively released or competitive binding with biotin receptor which was higher expressed on 4T1 tumor cells. The intracellular drugs, BPCD_І_ and free DOX degraded from conjugates, were exocytosed by MSCs via the passive diffusion or efflux by p-glycoprotein.
