# Supplementary figures and images for: Maximized nanodrug-loaded mesenchymal stem cells by a dual drug-loaded mode for the systemic treatment of metastatic lung cancer
Source: Drug Deliv. 2017 Sep 18;24(1):1372–83. doi: 10.1080/10717544.2017.1375580 (PMC8241180; doi:10.1080/10717544.2017.1375580)

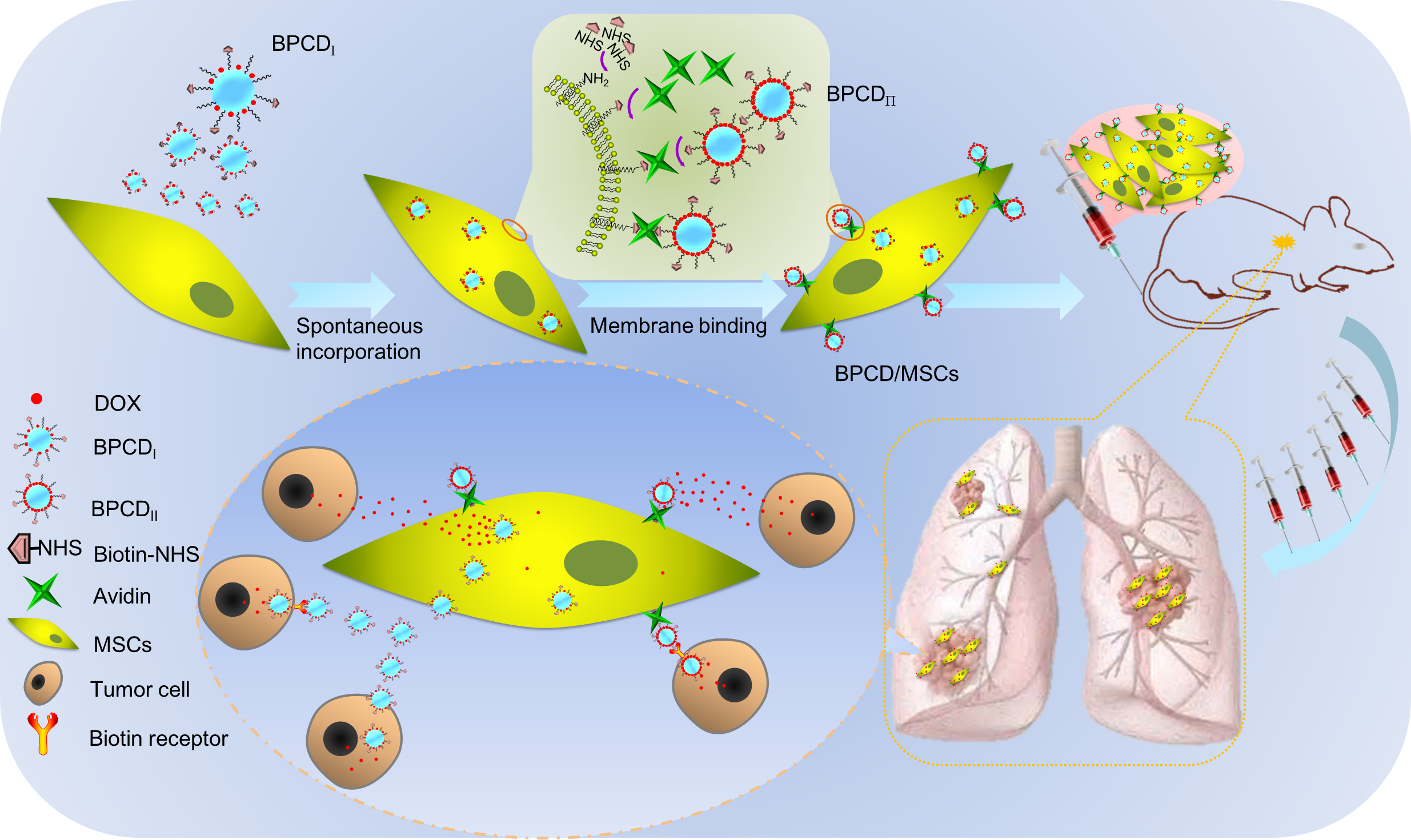

Supplement: IDRD_Jiang_et_al_Supplemental_Content.zip [file IDRD_A_1375580_SM4571.zip › Scheme 1.tif]
